# Addendum to the Previous Article: A Position Statement by Indian Association of Palliative Care

**DOI:** 10.4103/0973-1075.78456

**Published:** 2011

**Affiliations:** National Coordinator, Palliative Care Programme, Christian Medical Association of India (CMAI), New Delhi - 110058

Sir,

This is to request you to kindly make the following addendum to the previous article – “A position statement by Indian Association of Palliative Care”[1] and to please provide a link to the previous article. The main article remains as before.
